# Supplementary material for: A transposon insertion in the promoter of OsUBC12 enhances cold tolerance during japonica rice germination
Source: Nat Commun. 2024 Mar 13;15:2211. doi: 10.1038/s41467-024-46420-7 (PMC10937917; doi:10.1038/s41467-024-46420-7)
Supplement: Supplementary file 1 — Supplementary information [file 41467_2024_46420_MOESM1_ESM.pdf]

# A transposon insertion in the promoter of *OsUBC12* enhances cold tolerance during *japonica* rice germination

Zhang *et al*.

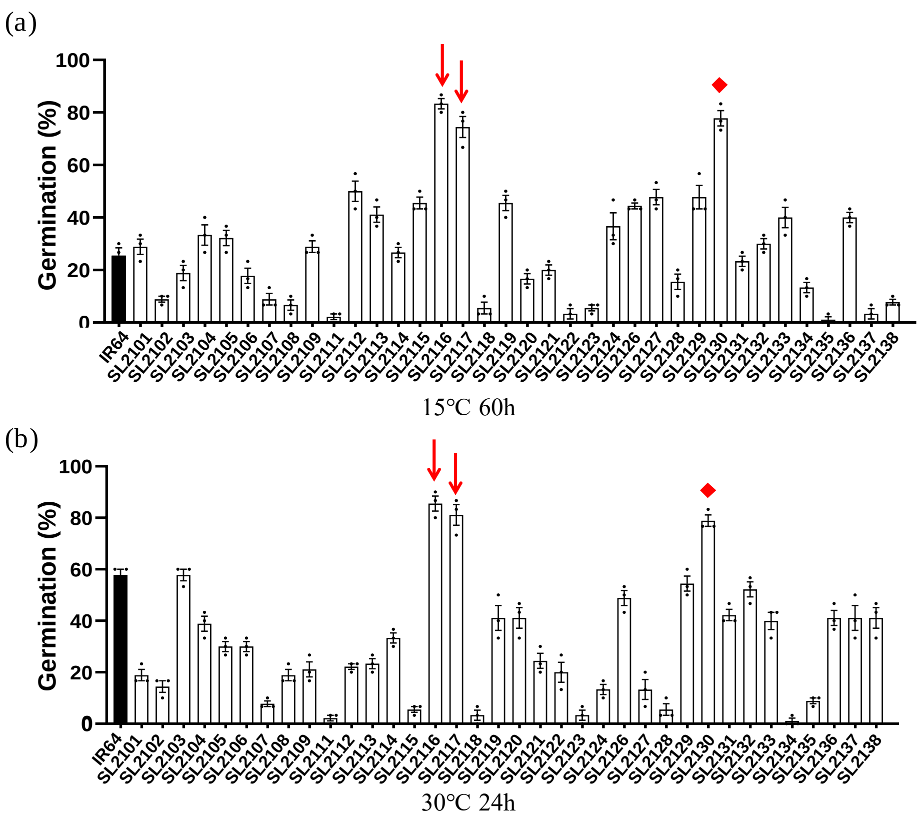


**Supplementary Figure 1. Germination rates of 36 chromosome segment substitution lines (CSSLs) following 60 h at 15 °C (a) or 24 h at 30 °C (b).** Values are mean ± standard error (SE) from three individual biological replicates (30 seeds per biological replicate). Source data are provided as a Source Data file.

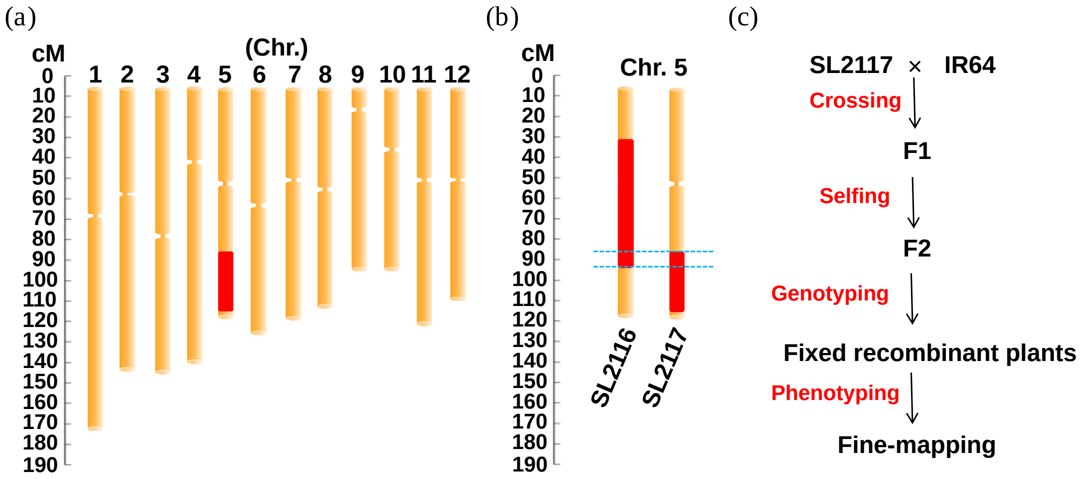


**Supplementary Figure 2. Low-temperature germination phenotype of SL2117 segregated as a semi-dominant trait of higher resistance to low temperature during germination.** (a) Graphical representation of the CSSL (SL2117) genotype. (b) Graphical genotypes of SL2116 and SL2117. Green region, genomic region from IR64; red region, genomic region from Koshihikari. (c) Schematic representation of the generation of an $F_2$ population from SL2117 × IR64.

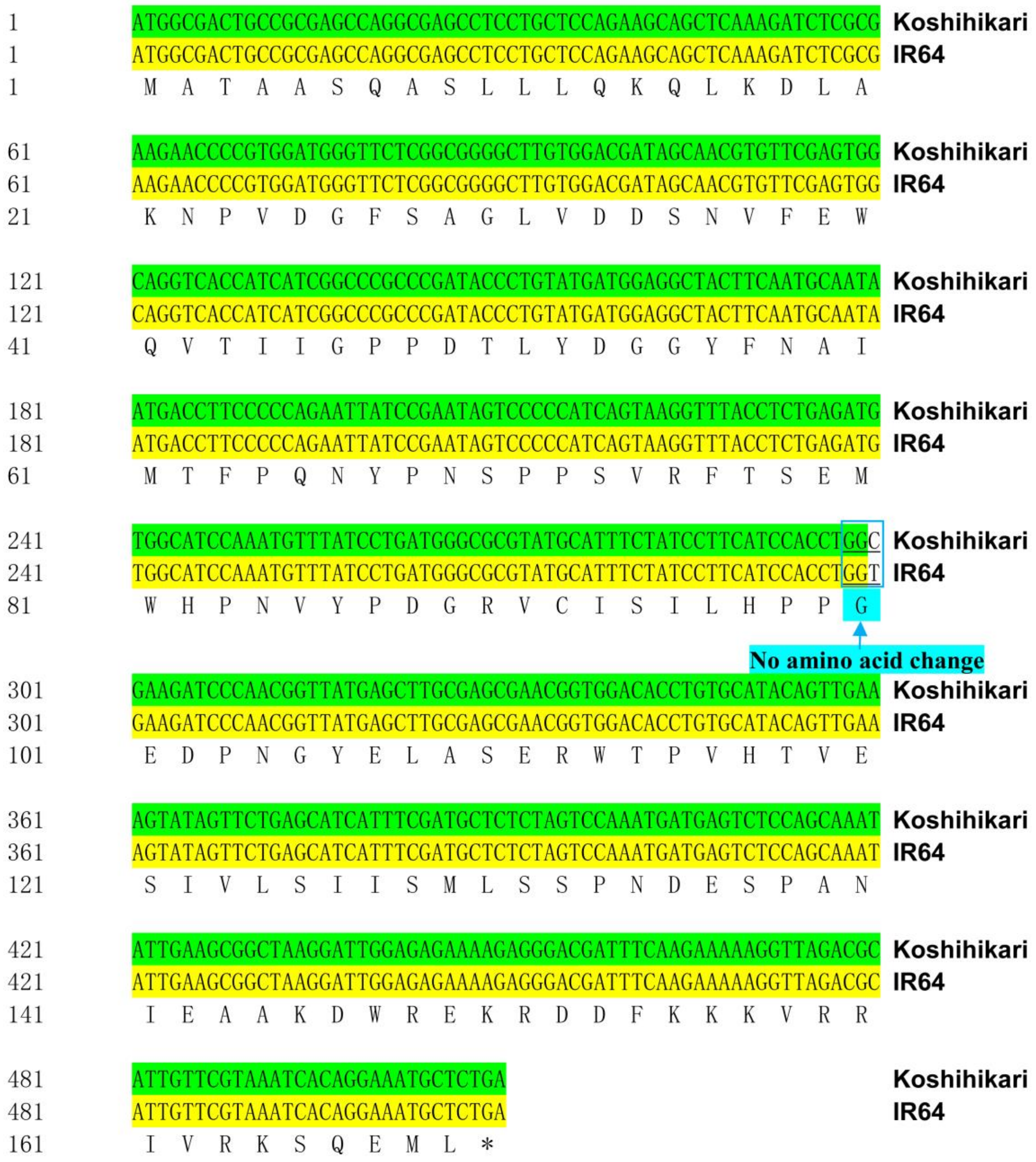


**Supplementary Figure 3. Sequence comparison of the *OsUBC12* open reading frame between *japonica* Koshihikari and *indica* IR64.** The red box indicates a nucleotide substitution.

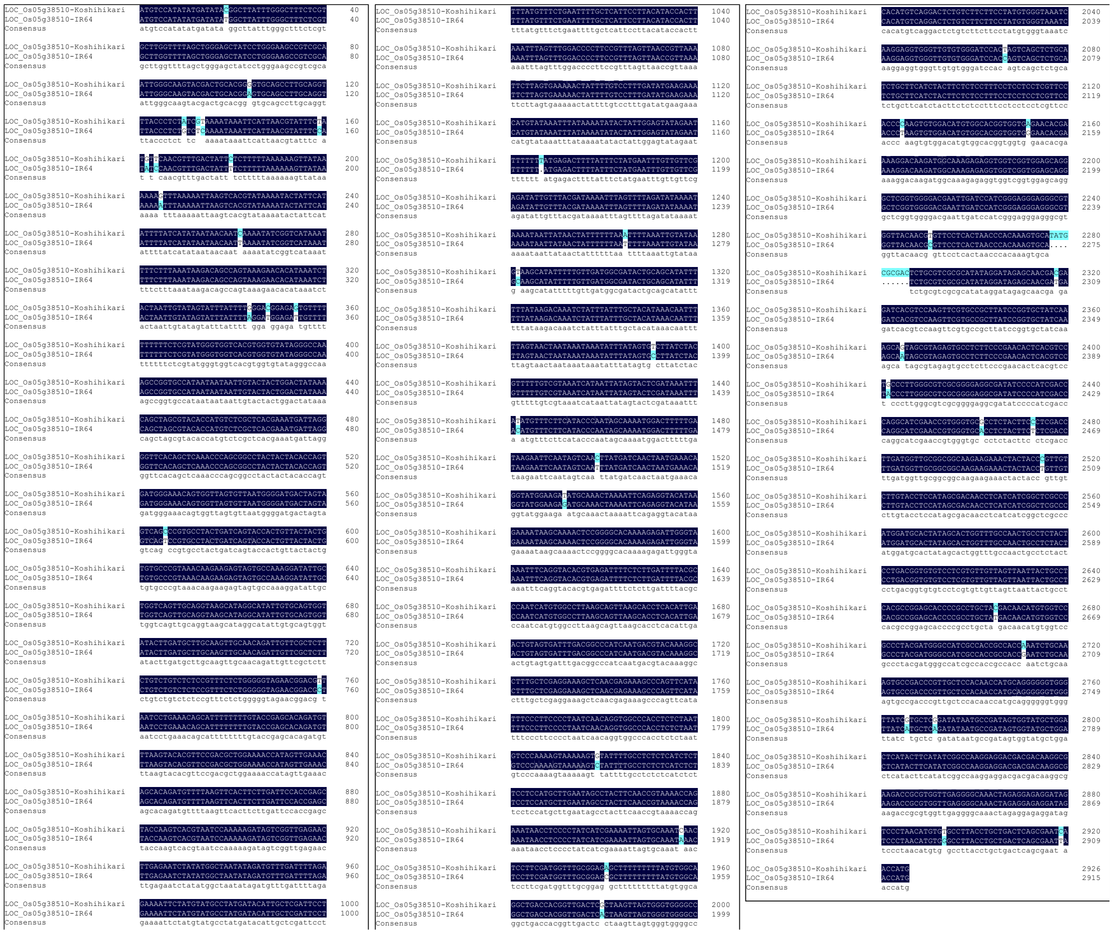

**Supplementary Figure 4. The genomic homologies of *LOC_Os05g38510* between Koshihikari and IR64.**

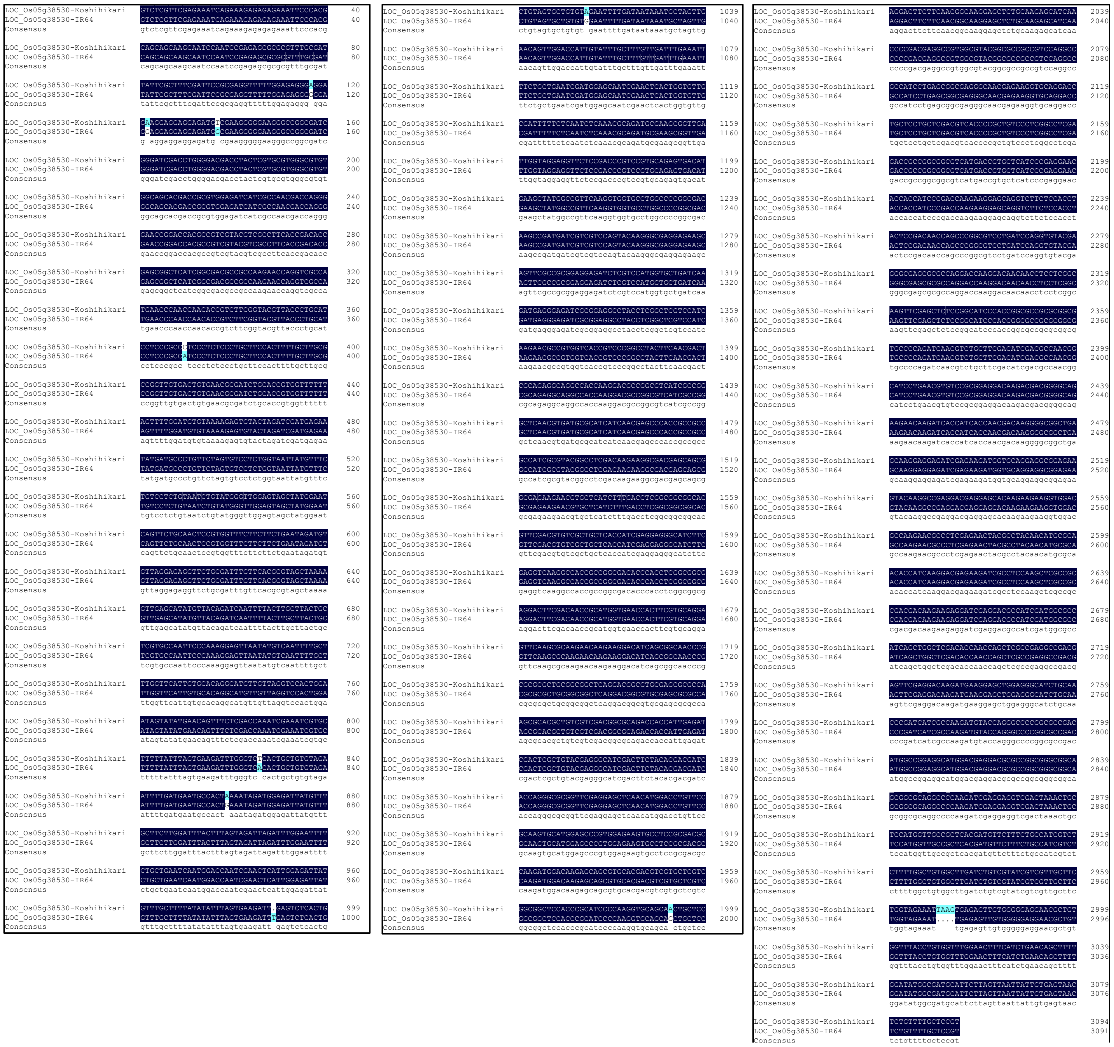

**Supplementary Figure 5. The genomic homologies of *LOC_Os05g38530* between Koshihikari and IR64.**

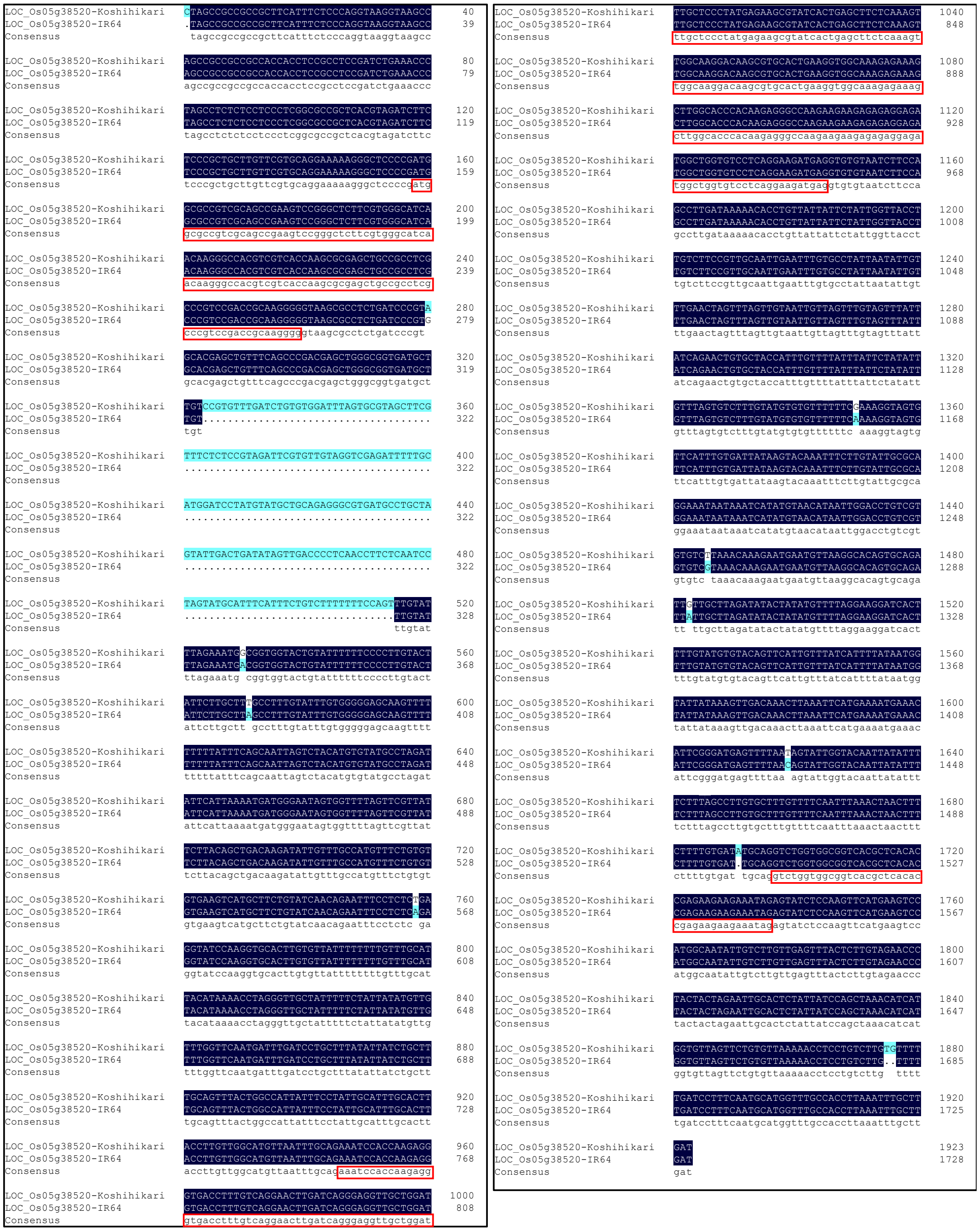

**Supplementary Figure 6. The genomic homologies of *LOC_Os05g38520* between Koshihikari and IR64.** All exons of *LOC_Os05g38520* were indicated by the red box.

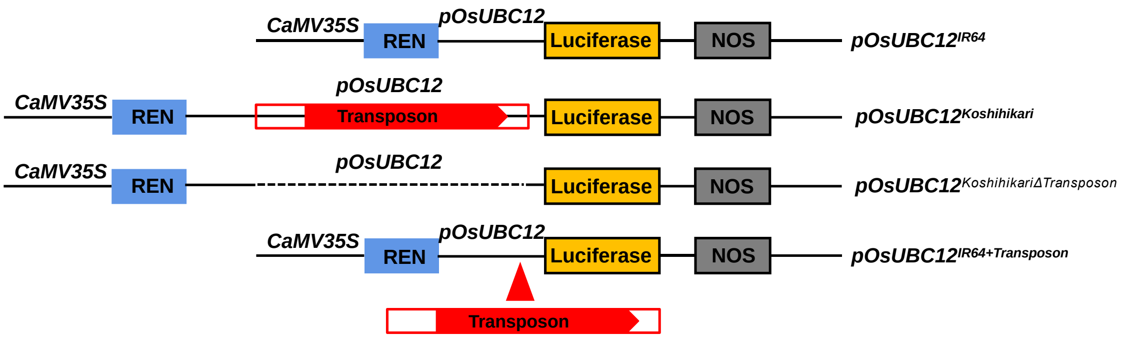


**Supplementary Figure 7. Schematic representation of the recombinant vectors p35S:REN–pOsUBC12$^{IR64}$:LUC, p35S:REN–pOsUBC12$^{Koshihikari}$:LUC, p35S:REN–pOsUBC12$^{Koshihikari\,\Delta\,Transposon}$:LUC, or p35S:REN–pOsUBC12$^{IR64+Transposon}$:LUC used in the dual-luciferase reporter assay.**

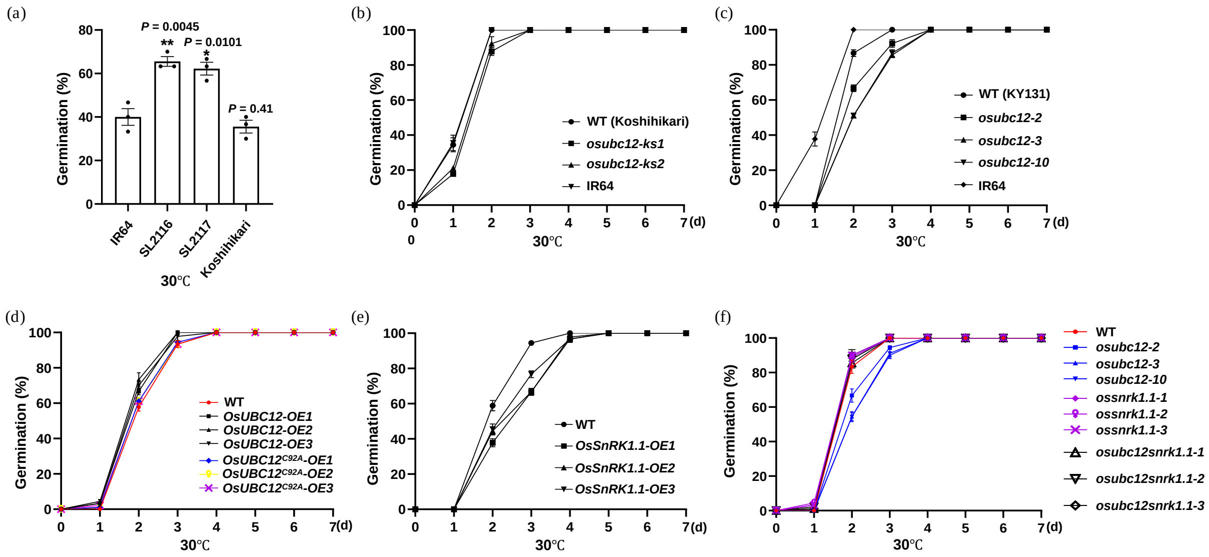


**Supplementary Figure 8. Germination rates of SL2116, SL2117, *osubc12-ks* mutants, *osubc12* mutants, *OsUBC12-OE*, *OsUBC12$^{C92A}$-OE*, *OsSnRK1.1-OE* lines, *ossnrk1.1* mutants and *osubc12snrk1.1* double mutants at 30 °C.** (a) Germination rates of SL2116 and SL2117 after 24 h at 30 °C. The Koshihikari and IR64 were used as controls. Values are means ± SE from three individual biological replicates (30 seeds per biological replicate). The data were statistically analyzed using two-tailed Student's *t*-test (*$P < 0.05$, **$P < 0.01$). (b) Time-course germination of *osubc12-ks* mutants at 30 °C. The WT (Koshihikari) and IR64 were used as controls. All values are means ± SE from three individual biological replicates (30 seeds per biological replicate). (c) Time-course germination of *osubc12* mutants at 30 °C. The WT (KY131) and IR64 were used as controls. All values are means ± SE from three individual biological replicates (30 seeds per biological replicate). (d) Time-course germination of WT (KY131), *OsUBC12-OE* and *OsUBC12$^{C92A}$-OE* lines at 30 °C. All values are means ± SE from three individual biological replicates (30 seeds per biological replicate). (e) Time-course germination of WT (KY131) and *OsSnRK1.1-OE* lines at 30 °C. All values are means ± SE from three individual biological replicates (30 seeds per biological replicate). (f) Time-course germination analysis of WT (KY131), *osubc12* mutants, *ossnrk1.1* mutants and *osubc12snrk1.1* double mutants at 30 °C. All values are means ± SE from three individual biological replicates (30 seeds per biological replicate). Source data are provided as a Source Data file.

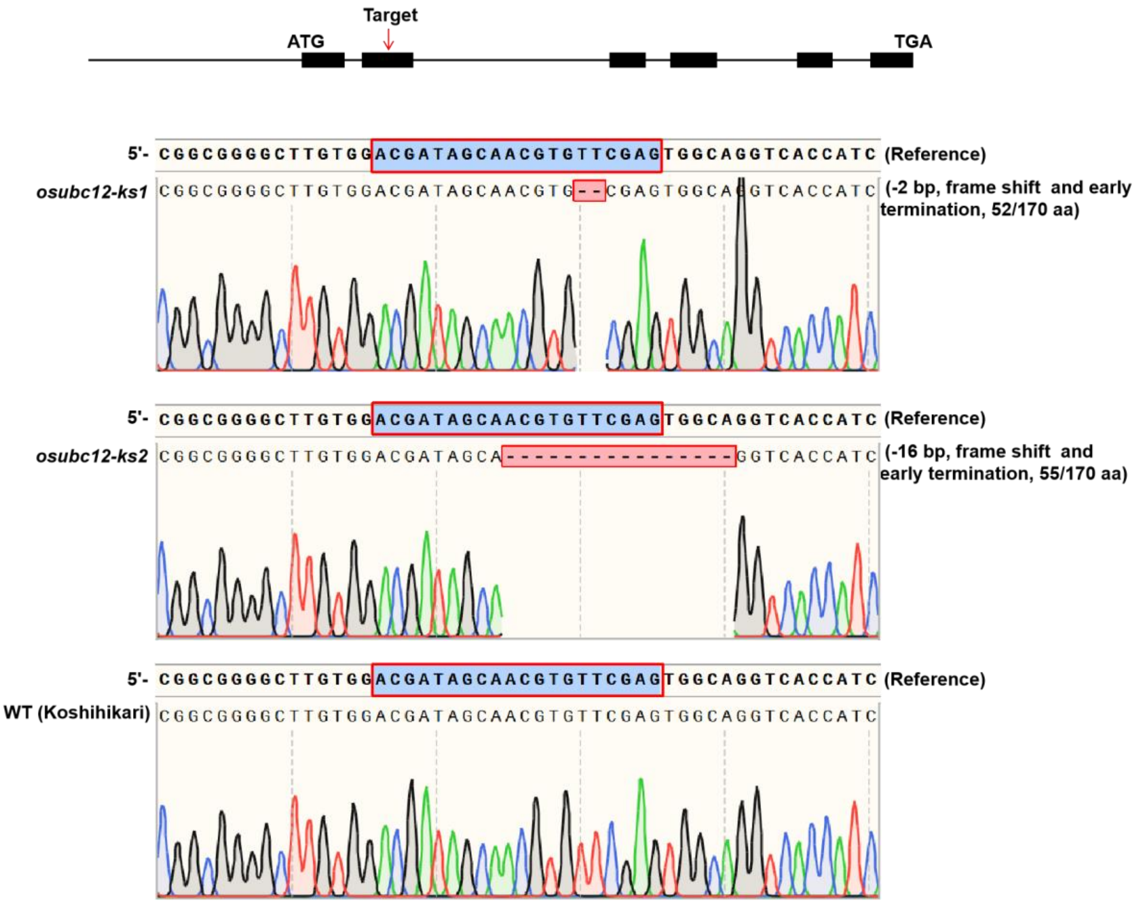


**Supplementary Figure 9. Identification of *osubc12-ks* mutants.** The *OsUBC12* target sequences are indicated by red boxes.

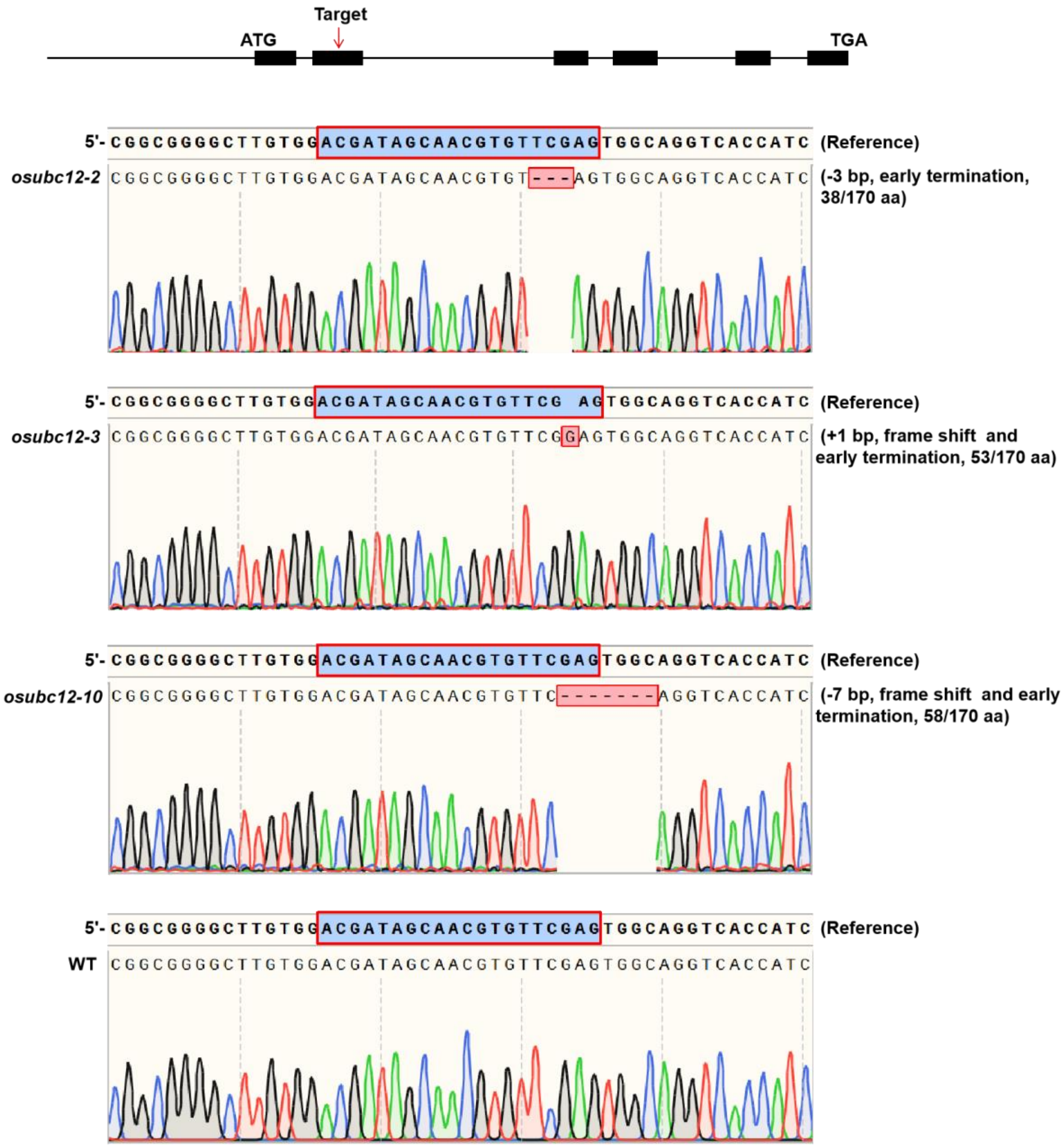


**Supplementary Figure 10. Identification of *osubc12* mutants.** The *OsUBC12* target sequences are indicated by red boxes.

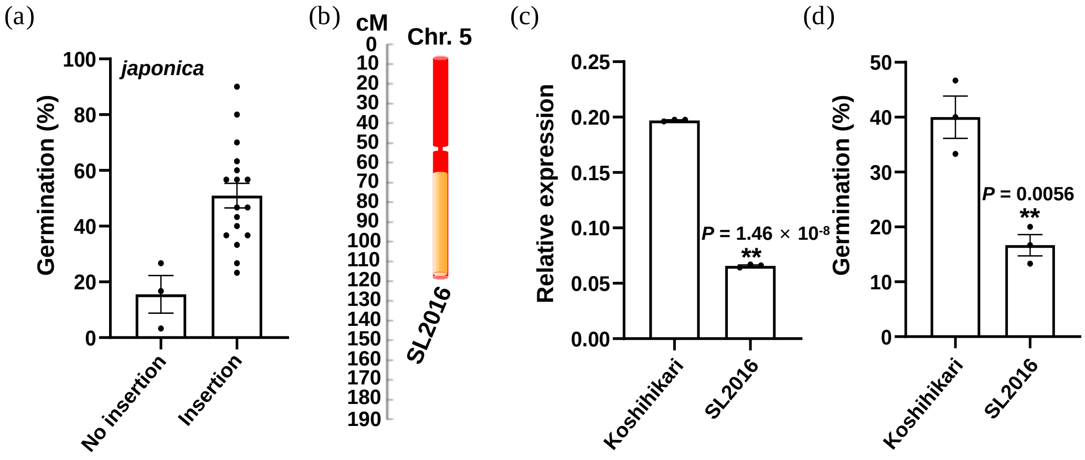


**Supplementary Figure 11. The low-temperature germinability of two groups (transposons + vs -) of *japonica*, and SL2016.** (a) Germination rates of two groups (transposons + vs -) of *japonica* after 60 h at 15 °C. Values are means ± SE from three individual replicates ($n = 3$) of *japonica* (transposons-) group or seventeen individual replicates ($n = 17$) of *japonica* (transposons+) group. (b) Graphical genotypes of SL2016. Green region, genomic region from IR64; red region, genomic region from Koshihikari. (c) Relative expression of *OsUBC12* in SL2016 seeds. Values are means ± SE from three individual replicates ($n = 3$). The housekeeping gene *OsUBQ5* was used as an internal control to normalize the data. The data were statistically analyzed using two-tailed Student's *t*-test ($*P < 0.05$, $**P < 0.01$). (d) Germination rates of SL2016 after 60 h at 15 °C. Values are means ± SE from three individual biological replicates (30 seeds per biological replicate). The data were statistically analyzed using two-tailed Student's *t*-test ($*P < 0.05$, $**P < 0.01$). Source data are provided as a Source Data file.

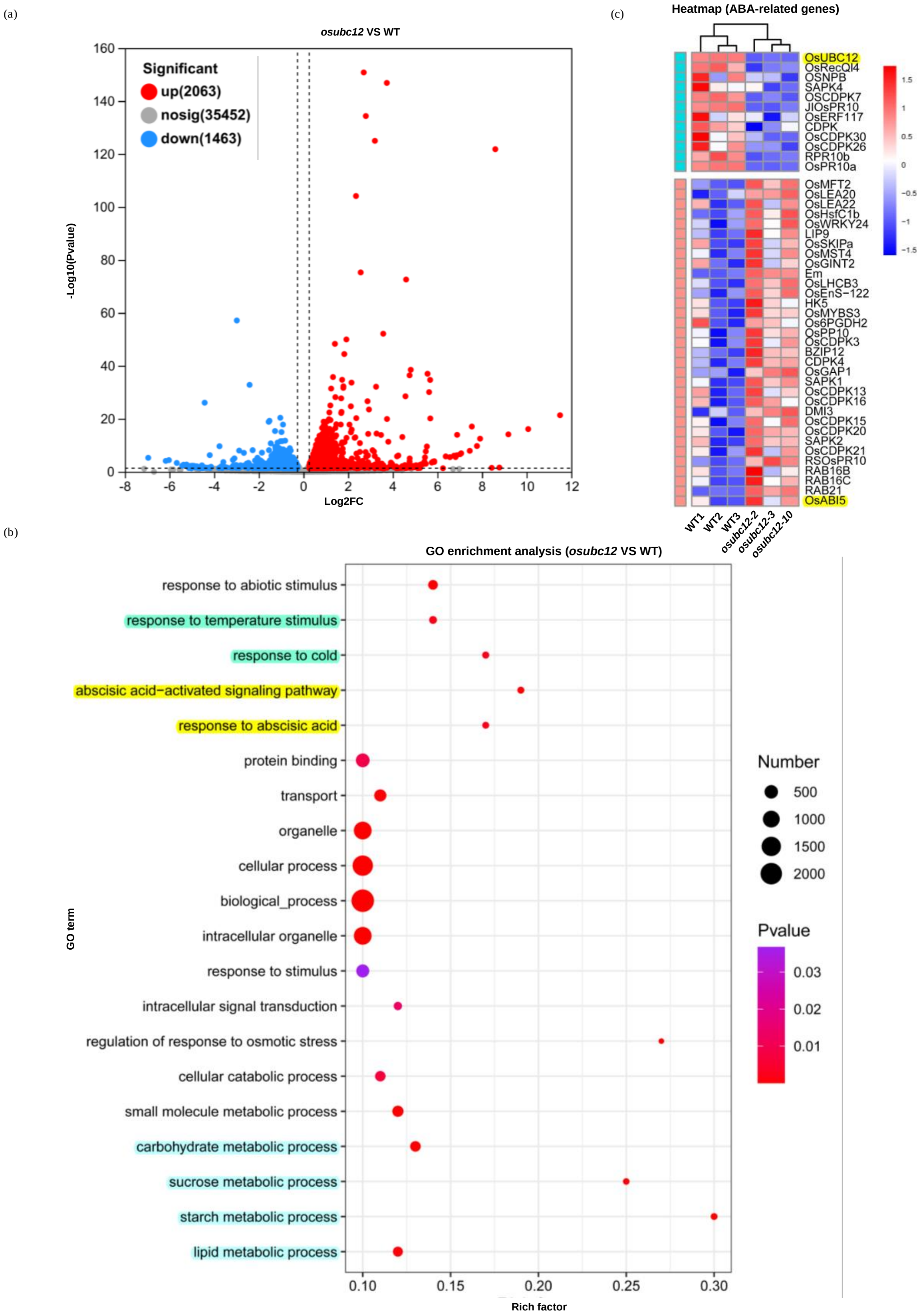


**Supplementary Figure 12. Volcano plots, GO enrichment and heat map**

**analysis of the significantly differentially expressed genes in *osubc12* mutants vs. WT (KY131) seeds germinated at low temperature (15 °C) detected by RNA-seq analysis.** (a) Volcano plots of the significantly differentially expressed genes in *osubc12* mutants vs. WT (KY131) seeds germinated at low temperature (15 °C). (b) GO enrichment analysis of the significantly differentially expressed genes in *osubc12* mutants vs. WT (KY131) germinated at low temperature (15 °C). (c) Heat map of microarray expression profiles for ABA-related genes in *osubc12* mutants vs. WT (KY131) germinated at low temperature (15 °C). The color scale represents the log2 (FPKM+1) and then standardized by “scale()” in R script.

(a)

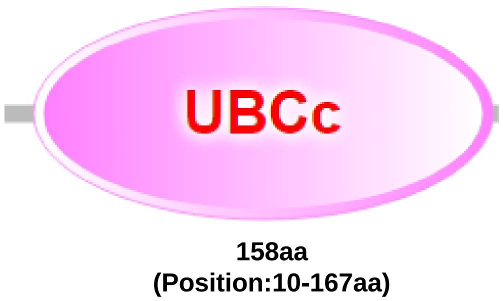


(b)

```
OsUBC12     ..................................MATAAS      6
AtUBC7      MCSHNENIDSRKFTKSEPCHIKKISISILSISLSSSSMAS     40
AtUBC27     .......................................M      1
OsUBC5b     ........................................      0
Consensus

OsUBC12     QASLLLQKQLKDLAKNPVDGFSAGLVDDSNVFEWQVTIIG     46
AtUBC7      QASLLLQKQLKDLCKHPVDGFSAGLVDEKNIFEWSVTIIG     80
AtUBC27     IDFSRIQKELQDCERNQDSSGIRVCPKSDNLTRLTGTIPG     41
OsUBC5b     MASKRILKELKDLQKDPPTS.CSAGPVAEDMFHWQATLMG     39
Consensus          k l d                        t  g

OsUBC12     PPDTLYDGGYFNAIMTFPQNYPNSPPSVRFTSEMWHPNVY     86
AtUBC7      PPDTLYEGGFFNAIMTFPQNYPNSPPTVRFTSDMWHPNVY    120
AtUBC27     PIGTPYEGGTFQIDITMPDGYPFEPPKMQFSTKVWHPNIS     81
OsUBC5b     PSDSPYAGGVFLVTIHFPPDYPFKPPKVALKTKVFHPNIN     79
Consensus   p    y gg f      p   yp  pp        hpn

OsUBC12     P.DGRVCISILHPPGEDPNGYELASERWTPVHTVESIVLS    125
AtUBC7      S.DGRVCISILHPPGDDPSGYELASERWTPVHTVESIMLS    159
AtUBC27     SQSGAICLDILK.............DQWSPALTLKTALVS    108
OsUBC5b     S.NGSICLDILK.............EQWSPALTISKVLLS    105
Consensus      g  c  il                w p  t      s

OsUBC12     IISMLSSPNDESPANIEAAKDWREKRDDFKKKVRRIVRKS    165
AtUBC7      IISMLSGPNDESPANVEAAKEWRDKRDEFKKKVSRCVRKS    199
AtUBC27     IQALLSAPEPKDPQDAVVAEQYMKNYQVFVSTARYWTETF    148
OsUBC5b     ICSLLTDPNPDDPLVPEIAHMYKTDRAKYESTARSWTQKY    145
Consensus   i   l  p    p     a

OsUBC12     QEML....................................    169
AtUBC7      QEMF....................................    203
AtUBC27     AKKSSLEEKVKRLVEMGFGDAQVRSAIESSGGDENLALEK    188
OsUBC5b     AMG.....................................    148
Consensus

OsUBC12     ...                                         169
AtUBC7      ...                                         203
AtUBC27     LCS                                         191
OsUBC5b     ...                                         148
Consensus
```

**Supplementary Figure 13. The protein structure and sequence alignment of OsUBC12.** (a) Predicted domains in OsUBC12 protein by SMART website (http://smart.embl_x0002_heidelberg.de/). A UBC domain (pink) is predicted in OsUBC12. aa, amino acid. (b) Alignment of Arabidopsis UBC7 (AtUBC7, Q42540), Arabidopsis UBC27 (AtUBC27, Q9FI61) and rice UBC5a (OsUBC5a, Q8S919). The active-site cysteine residue cysteine of the UBC domain is indicated by the red arrow, and the UBC domain is shown in red box.

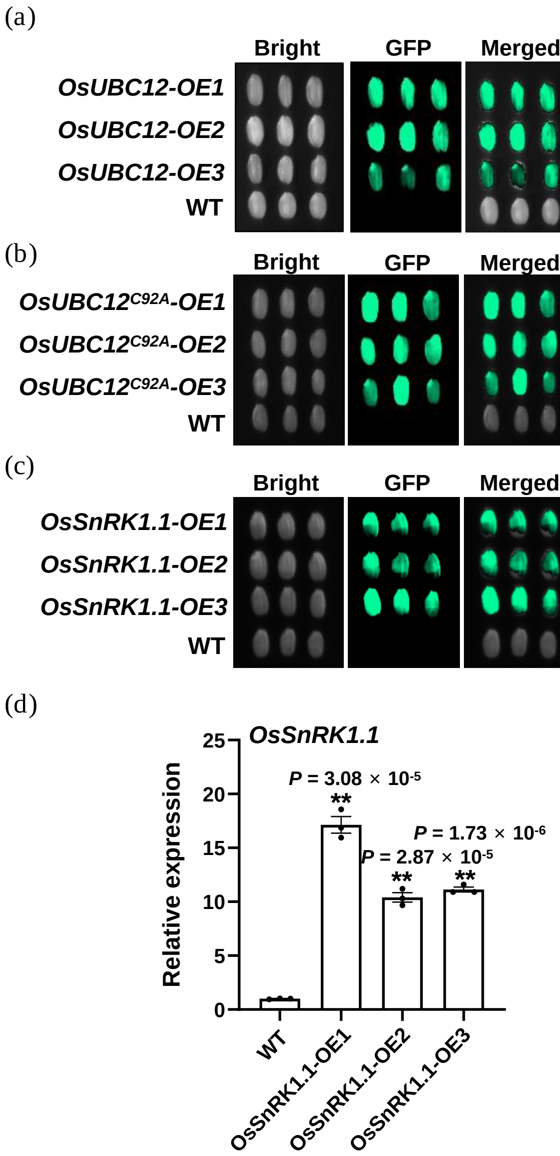


**Supplementary Figure 14. Identification of *OsUBC12-OE*, *OsUBC12$^{C92A}$-OE* and *OsSnRK1.1-OE* lines.** (a) Identification of *OsUBC12-OE* lines by fluorescence screening. Scale bars, 0.5 cm. (b) Identification of *OsUBC12$^{C92A}$-OE* lines by fluorescence screening. Scale bars, 0.5 cm. (c) Identification of *OsSnRK1.1-OE* lines by fluorescence screening. Scale bars, 0.5 cm. (d) Relative expression of *OsSnRK1.1* in seeds of *OsSnRK1.1-OE* lines. The expression level of the control sample (WT, KY131) was set to 1. Values are means ± SE from three individual replicates ($n = 3$). The housekeeping gene *OsUBQ5* was used as an internal control to normalize the data. The data were statistically analyzed using two-tailed Student's *t*-test (*$P < 0.05$, **$P < 0.01$). Source data are provided as a Source Data file.

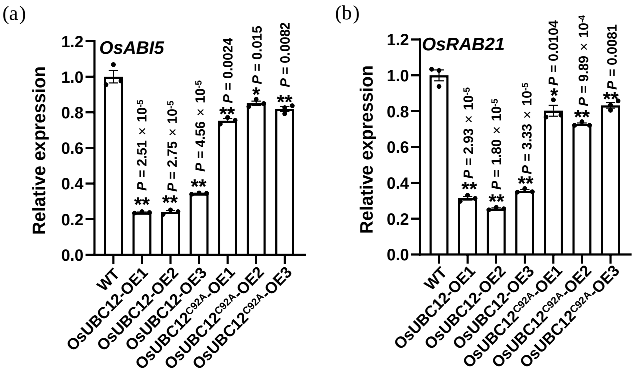


**Supplementary Figure 15. Relative expression of *OsABI5* (a) and *OsRAB21* (b) in seeds of *OsUBC12-OE* and *OsUBC12*$^{C92A}$*-OE* lines.** The expression level of the control samples (WT, KY131) was set to 1. Values are means ± SE from three individual replicates ($n = 3$). The housekeeping gene *OsUBQ5* was used as an internal control to normalize the data. The data were statistically analyzed using two-tailed Student's *t*-test (*$P < 0.05$, **$P < 0.01$). Source data are provided as a Source Data file.

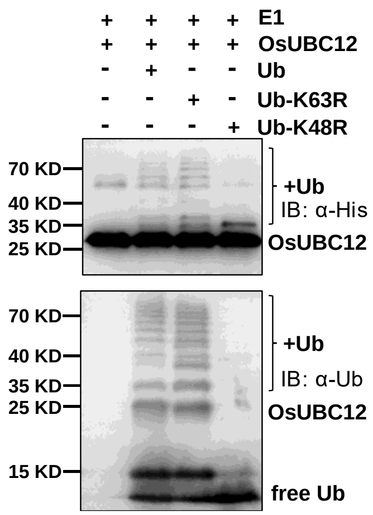


**Supplementary Figure 16. The polyubiquitination type of OsUBC12.** Ubiquitin and two mutated Ub variants (Ub-K63R and Ub-K48R) were used in the reactions. Poly-Ub conjugates were detected using anti-His and anti-Ub antibodies. Source data are provided as a Source Data file.

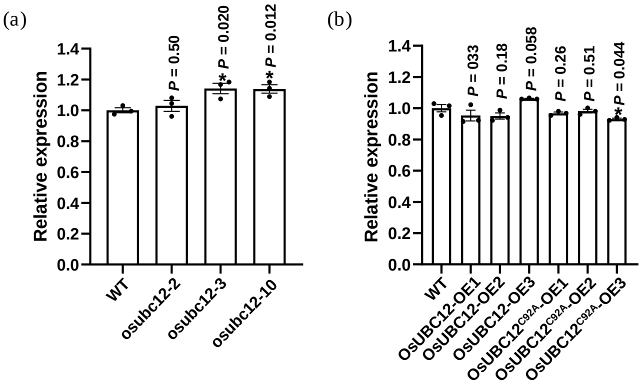


**Supplementary Figure 17. Relative expression of *OsSnRK1.1* in seeds of *osubc12* mutants (a), *OsUBC12-OE*, *OsUBC12$^{C92A}$-OE* lines (b).** The expression level of the control sample (WT, KY131) was set to 1. Values are means ± SE from three individual replicates ($n = 3$). The housekeeping gene *OsUBQ5* was used as an internal control to normalize the data. The data were statistically analyzed using two-tailed Student's *t*-test (*$P < 0.05$, **$P < 0.01$). Source data are provided as a Source Data file.

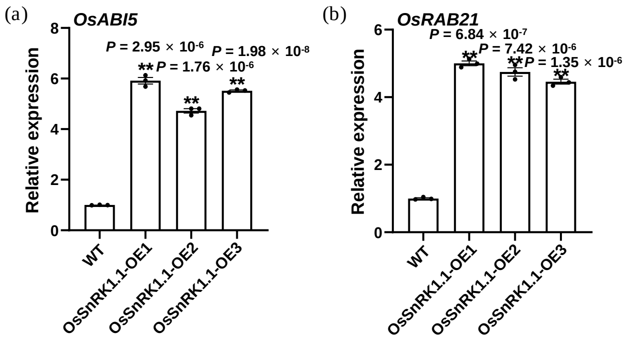


**Supplementary Figure 18. Relative expression of *OsABI5* (a) and *OsRAB21* (b) in seeds of *OsSnRK1.1-OE* lines.** The expression level of the control samples (WT, KY131) was set to 1. Values are means ± SE from three individual replicates ($n = 3$). The housekeeping gene *OsUBQ5* was used as an internal control to normalize the data. The data were statistically analyzed using two-tailed Student's *t*-test (*$P < 0.05$, **$P < 0.01$). Source data are provided as a Source Data file.

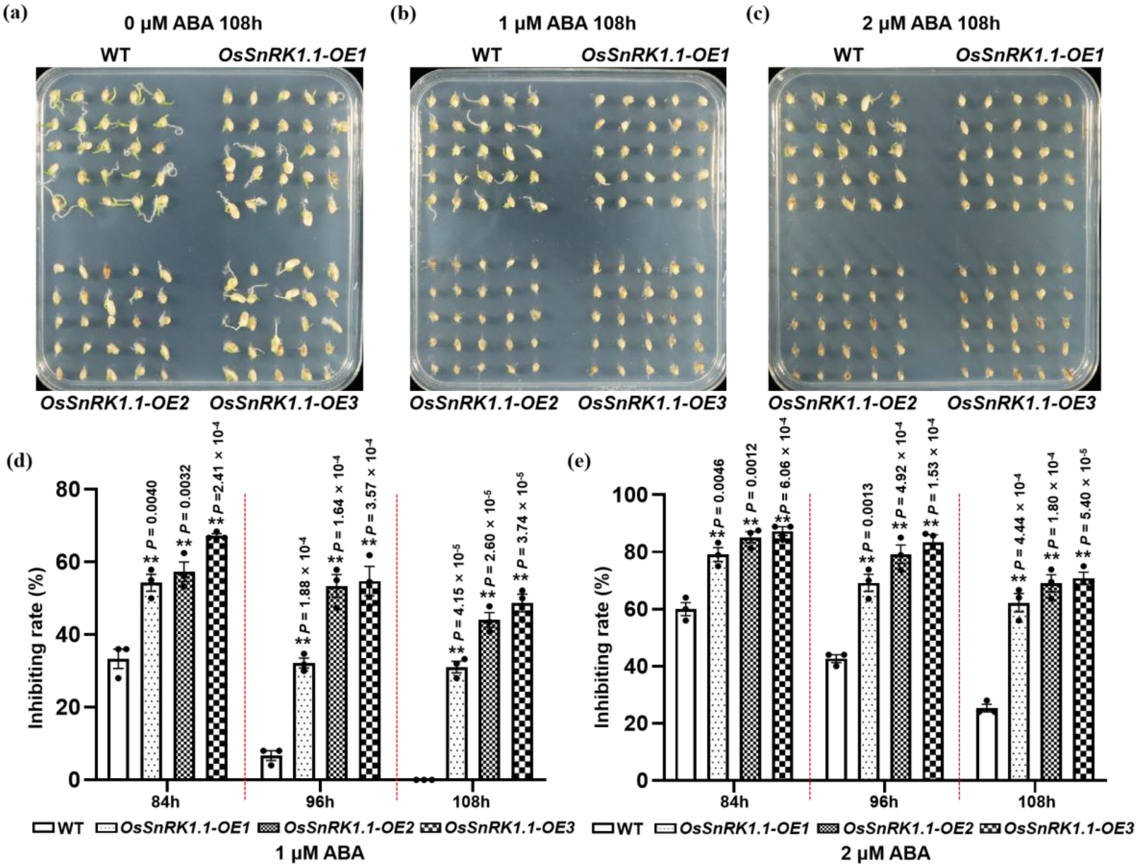


**Supplementary Figure 19. OsSnRK1.1 functions opposite to OsUBC12 in ABA signaling.** (a), (b) Germination performance of WT (KY131) and *OsSnRK1.1-OE* seeds after 108 h on ½-MS agar medium containing 0 µM (a), 1 µM (b) or 2 µM (c) ABA. (d), (e) The germination inhibition rates of WT (KY131) and *OsSnRK1.1-OE* seeds under 1 µM (d) or 2 µM (e) ABA at three timepoints. Values are means ± SE from three individual biological replicates (25 seeds per biological replicate). The data were statistically analyzed using two-tailed Student's *t*-test (*$P < 0.05$, **$P < 0.01$). Source data are provided as a Source Data file.

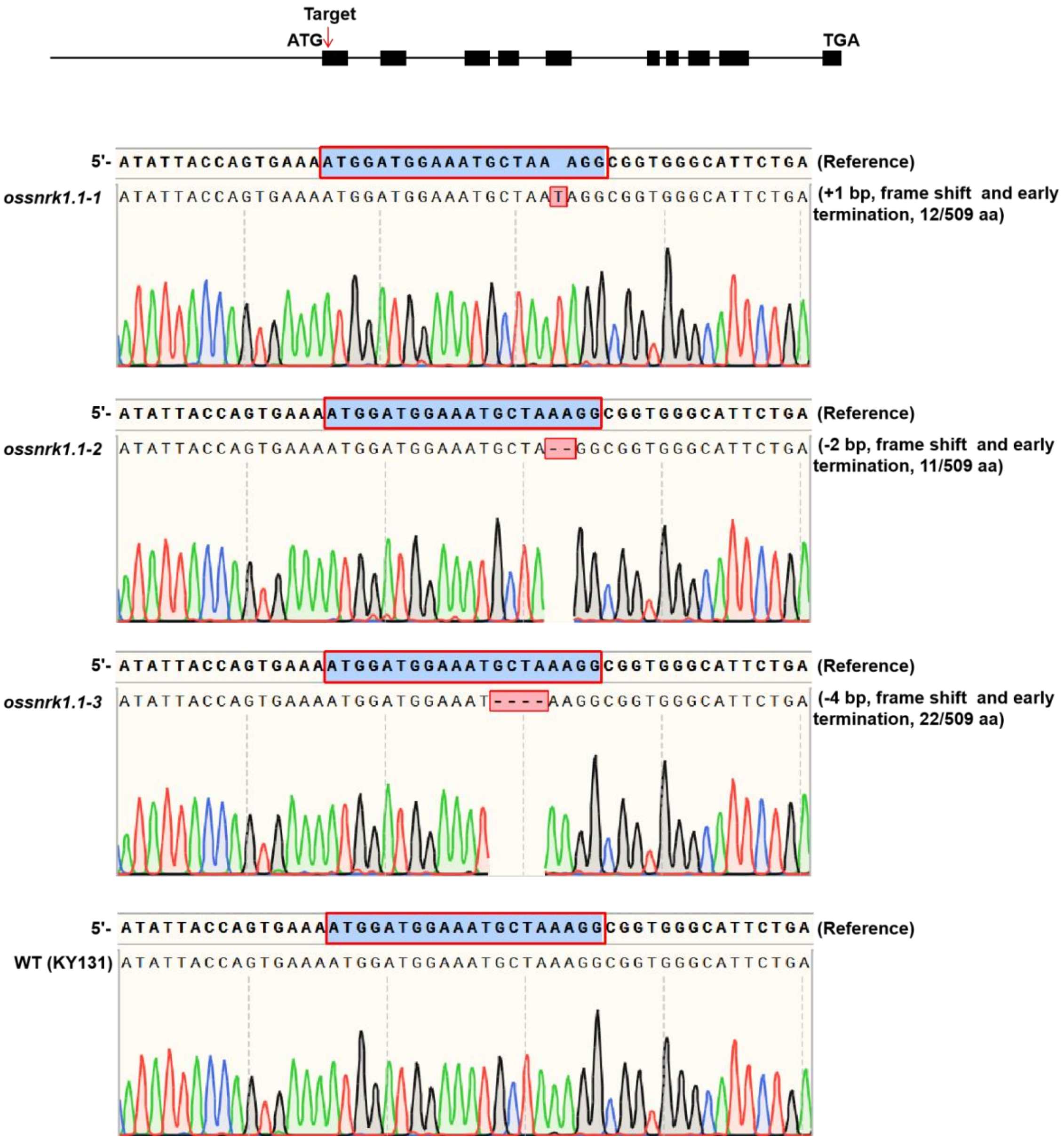


**Supplementary Figure 20. Identification of *ossnrk1.1* mutants.** The *OsSnRK1.1* target sequences are indicated by red boxes.

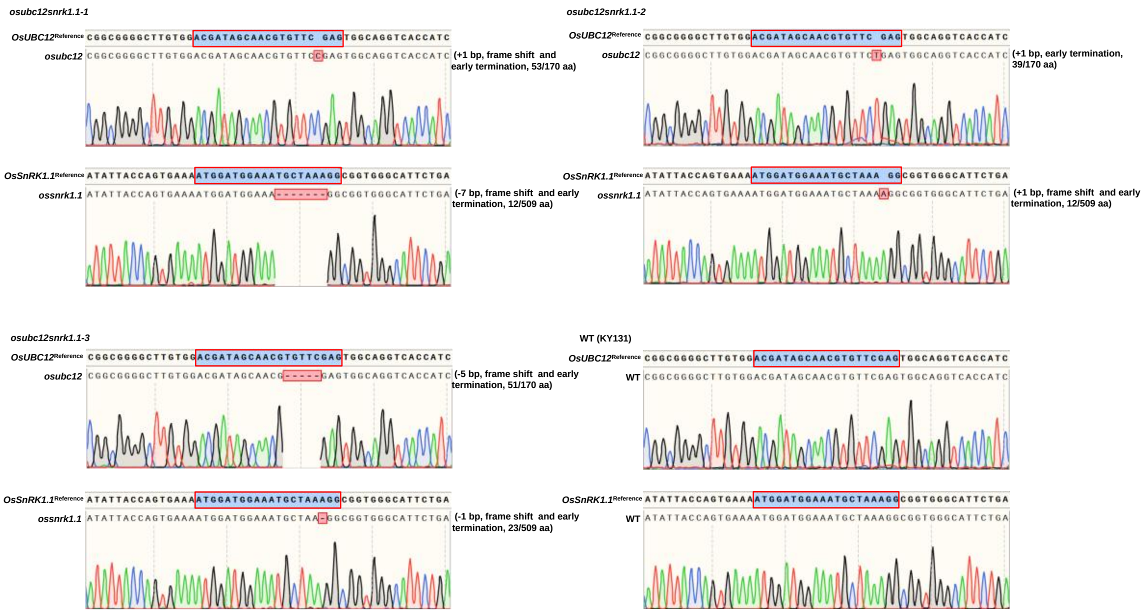


**Supplementary Figure 21. Identification of *osubc12snrk1.1* double mutants.** The *OsUBC12* and *OsSnRK1.1* target sequences are indicated by red boxes, respectively.

(a)

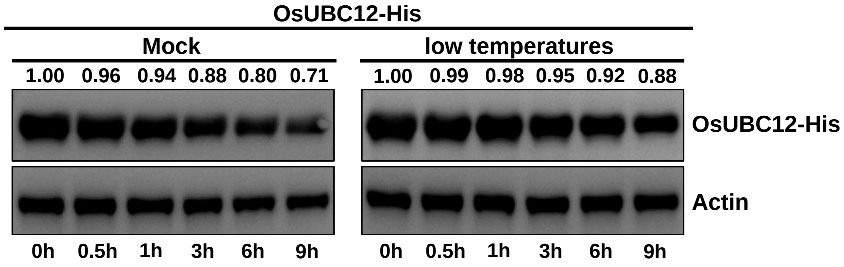


(b)

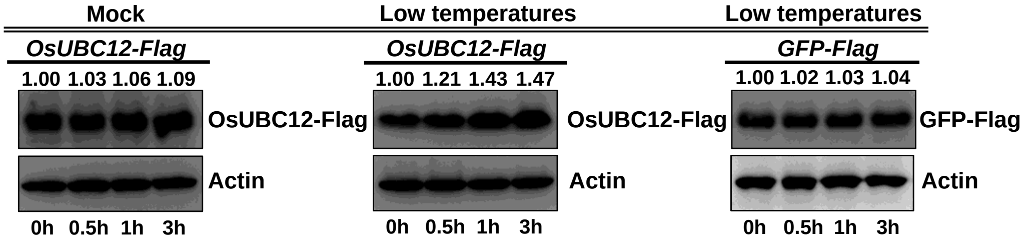


**Supplementary Figure 22. Low temperature could promote the accumulation of OsUBC12 protein *in vitro* and *in vivo*.** (a) Cell-free degradation assays of OsUBC12-His incubated with protein extracts from mock-treated WT (KY131) seeds or WT seeds (KY131) following a 12-h treatment with low temperature (15 °C). Protein extracts from mock-treated WT (KY131) or low temperature-treated WT (KY131) were incubated with OsUBC12-His for the indicated durations. OsUBC12-His levels were visualized by immunoblotting using an anti-His antibody. OsActin was used as the loading control. To quantify relative protein band intensity, the protein band at 0 h was set to 1.00. (b) *In vivo* protein accumulation analysis of OsUBC12-Flag in rice protoplasts. Recombine plasmid *OsUBC12-Flag* were transiently expressed in the protoplasts by PEG-mediated transfection. Following 12 h incubation in the dark at 28 °C, the protoplasts were subjected to mock (28 °C) or low temperature (15 °C) treatment, and samples were taken according to time points. The protein extracts were subjected to immunoblotting with anti-FLAG antibody. OsActin was used as the loading control. The expression of *GFP-Flag* under low temperature was used as a reference. To quantify relative protein band intensity, the protein band at 0 h was set to 1.00. Source data are provided as a Source Data file.

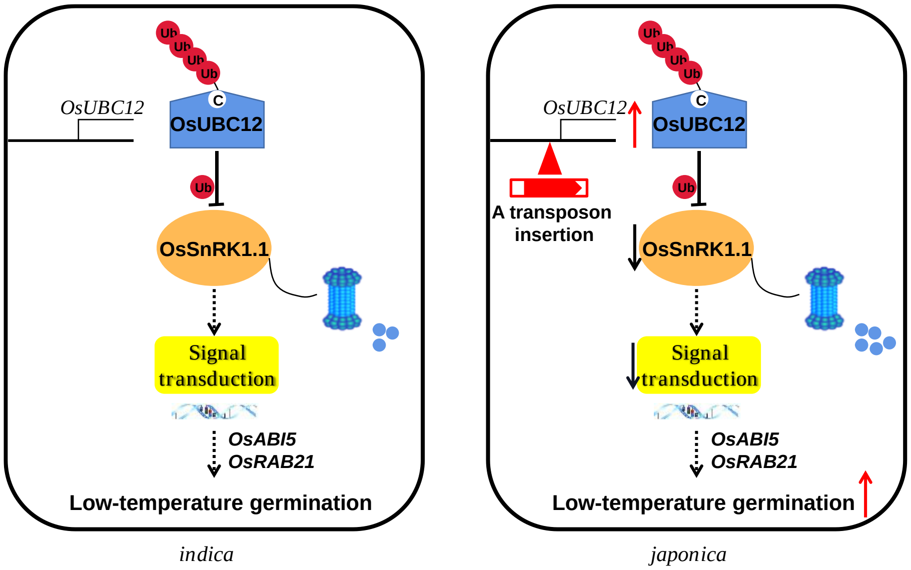


**Supplementary Figure 23. Proposed working model of OsUBC12 in *indica* and *japonica* rice.** According to our model, OsSnRK1.1 functions as a downstream key regulator to enhance ABA signaling by up-regulating the expression of ABA signaling-related genes such as *OsABI5* and *OsRAB21*, thus inhibiting LTG. OsUBC12, an E2 enzyme for Lys48-linked polyubiquitination, recruits and degrades OsSnRK1.1. Compared with *indica* rice, a transposon insertion in the *japonica OsUBC12* promoter activates the expression of *OsUBC12*. Increased OsUBC12 levels further promote the degradation of OsSnRK1.1, thereby weakening OsSnRK1.1-regulated ABA signaling and enhancing low-temperature germinability in *japonica* rice.

**Supplementary Table 1. Statistical analysis of the LTG phenotypes of $F_2$ plants using a $\chi^2$ test.**

| Resistant | Semi-resistant | Sensitive | $\chi^2$ | P value |
|---|---|---|---|---|
| 98 | 182 | 94 | 0.90 | 0.64 |

**Supplementary Table 2. The presence or absence of the transposon in the *OsUBC12* promoter is shown in a rice diversity panel.**

| **Group** | **No insertion** | **Insertion** |
|---|---|---|
| GJ-trp | 1 | 5 |
| GJ-sbtrp | 1 | 5 |
| GJ-tmp | 1 | 6 |
| XI-1A | 7 | 0 |
| XI-1B | 11 | 0 |
| XI-2 | 7 | 0 |
| XI-3 | 9 | 0 |
| XI-adm | 1 | 0 |
| Xian | 2 | 0 |
| cA(Aus) | 7 | 0 |
| cB(Bas) | 3 | 3 |
| Wild | 6 | 0 |
| *Fst* | 0.976 | |

GJ-trp, tropical *Geng/Japonica*; GJ-sbtrp, subtropical *Geng/Japonica*; GJ-tmp, temperate *Geng/Japonica*; XI-1A, *Xian/Indica*-1A group; XI-1B, *Xian/Indica*-1B group; XI-2, *Xian/Indica*-2 group; XI-3, *Xian/Indica*-3 group; XI-adm, admixed *Xian/Indica* group. Xian, *Xian/Indica*; cA(Aus), circum-*Aus* group; cB(Bas), circum-*Basmati* group; Wild, wild rice. Subgroup information for each variety is from Zhang et al. 2022 [61]. All varieties and their associated subgroup, haplotype information are listed in Supplementary Data 3.

**Supplementary Table 3. The presence absence variation of the transposon in 197 Asian domesticated rice accessions. cA(Aus), circum-*Aus* group.**

| **Group** | **No insertion** | **Insertion** |
|---|---|---|
| indica | 134 | 1 |
| japonica | 30 | 28 |
| aus | 4 | 0 |

**Supplementary Table 4. Expressed sequence tags (ESTs) from partial candidate genes encoding proteins isolated in the yeast two-hybrid (Y2H) screen of OsUBC12.**

| Accession | Encoding protein | Clones |
|---|---|---|
| LOC_Os01g01700 | Similar to RING-box protein 1A (regulator of cullins 1a) | 1 |
| LOC_Os03g1798 | Serine/threonine protein kinase | 3 |
| LOC_Os05g3855 | Ubiquitin-conjugating enzyme 12 | 1 |
| LOC_Os02g2643 | WRKY transcription factor 42 | 2 |
| LOC_Os11g2580 | Uncharacterized protein | 1 |
| LOC_Os02g4808 | Extracellular kinase-1 | 1 |
