# Supplementary material for: A transposon insertion in the promoter of OsUBC12 enhances cold tolerance during japonica rice germination
Source: Nat Commun. 2024 Mar 13;15:2211. doi: 10.1038/s41467-024-46420-7 (PMC10937917; doi:10.1038/s41467-024-46420-7)
Supplement: Supplementary file 3 — Description of Additional Supplementary Files [file 41467_2024_46420_MOESM3_ESM.pdf]

Description of Additional Supplementary Files

Supplementary Data 1-6:

Supplementary Data 1. List of primers used in this study.

Supplementary Data 2. The polymorphisms in the promoter region of *OsUBC12* between Koshihikari and IR64.

Supplementary Data 3. List of varieties for each subgroup used in Supplemental Table 2.

Supplementary Data 4. All significantly differentially expressed genes in *osubc12* mutants vs. WT (KY131) seeds germinated at low temperature (15 °C) detected by RNA-seq analysis.

Supplementary Data 5. GO Analysis statistical table of *osubc12* mutants vs. WT (KY131) seeds germinated at low temperature (15 °C) detected by RNA-seq analysis.

Supplementary Data 6. A list of ABA-related genes in *osubc12* mutants vs. WT (KY131) seeds germinated at low temperature (15 °C) detected by RNA-seq analysis.
