# Supplementary material for: A transposon insertion in the promoter of OsUBC12 enhances cold tolerance during japonica rice germination
Source: Nat Commun. 2024 Mar 13;15:2211. doi: 10.1038/s41467-024-46420-7 (PMC10937917; doi:10.1038/s41467-024-46420-7)
Supplement: Supplementary file 10 — Reporting Summary [file 41467_2024_46420_MOESM10_ESM.pdf]

nature portfolio

Corresponding author(s): Jun Fang

Last updated by author(s): Feb 21, 2024

Reporting Summary

Nature Portfolio wishes to improve the reproducibility of the work that we publish. This form provides structure for consistency and transparency in reporting. For further information on Nature Portfolio policies, see our Editorial Policies and the Editorial Policy Checklist.

Statistics

For all statistical analyses, confirm that the following items are present in the figure legend, table legend, main text, or Methods section.

n/a	Confirmed	
☐	☒	The exact sample size (*n*) for each experimental group/condition, given as a discrete number and unit of measurement
☐	☒	A statement on whether measurements were taken from distinct samples or whether the same sample was measured repeatedly
☐	☒	The statistical test(s) used AND whether they are one- or two-sided<br>*Only common tests should be described solely by name; describe more complex techniques in the Methods section.*
☒	☐	A description of all covariates tested
☐	☒	A description of any assumptions or corrections, such as tests of normality and adjustment for multiple comparisons
☐	☒	A full description of the statistical parameters including central tendency (e.g. means) or other basic estimates (e.g. regression coefficient) AND variation (e.g. standard deviation) or associated estimates of uncertainty (e.g. confidence intervals)
☐	☒	For null hypothesis testing, the test statistic (e.g. *F*, *t*, *r*) with confidence intervals, effect sizes, degrees of freedom and *P* value noted<br>*Give P values as exact values whenever suitable.*
☒	☐	For Bayesian analysis, information on the choice of priors and Markov chain Monte Carlo settings
☒	☐	For hierarchical and complex designs, identification of the appropriate level for tests and full reporting of outcomes
☒	☐	Estimates of effect sizes (e.g. Cohen's *d*, Pearson's *r*), indicating how they were calculated

Our web collection on statistics for biologists contains articles on many of the points above.

Software and code

Policy information about availability of computer code

Data collection	The immunoblot analysis were acquired by Tanon-5200.
Data analysis	ImageJ software (http://imagej.nih.gov/ij/index.html) was used for band density analysis.<br>Data analysis were performed using Graphpad Prism V8.0.2 and Microsoft Excel.

For manuscripts utilizing custom algorithms or software that are central to the research but not yet described in published literature, software must be made available to editors and reviewers. We strongly encourage code deposition in a community repository (e.g. GitHub). See the Nature Portfolio guidelines for submitting code & software for further information.

Data

Policy information about availability of data

All manuscripts must include a data availability statement. This statement should provide the following information, where applicable:

- Accession codes, unique identifiers, or web links for publicly available datasets
- A description of any restrictions on data availability
- For clinical datasets or third party data, please ensure that the statement adheres to our policy

All data supporting the findings of this study are available in the main text and its supplementary information. Raw RNA sequencing data are available at the NCBI Sequence ReadArchive (SRA) under accession PRJNA1050330, and presented in Supplementary Data 4-6. Sequence data from this study can be accessed from the Rice Genome Annotation Project website (http://rice.plantbiology.msu.edu/) under the following accession numbers: OsUBC12 (LOC_Os05g38550), OsSnRK1.1

(LOC_Os03g17980), OsABI5 (LOC_Os01g64000), OsRAB21 (LOC_Os11g26790), and OsUBQ5 (LOC_Os01g22490). All the source data for graphs in Figures and Supplementary Information are presented in Source Data. Raw images of the western blots are provided in in Source Data. Source data are provided with this paper.

Research involving human participants, their data, or biological material

Policy information about studies with human participants or human data. See also policy information about sex, gender (identity/presentation), and sexual orientation and race, ethnicity and racism.

Reporting on sex and gender	N/A
Reporting on race, ethnicity, or other socially relevant groupings	N/A
Population characteristics	N/A
Recruitment	N/A
Ethics oversight	N/A

Note that full information on the approval of the study protocol must also be provided in the manuscript.

Field-specific reporting

Please select the one below that is the best fit for your research. If you are not sure, read the appropriate sections before making your selection.

☒ Life sciences ☐ Behavioural & social sciences ☐ Ecological, evolutionary & environmental sciences

For a reference copy of the document with all sections, see nature.com/documents/nr-reporting-summary-flat.pdf

Life sciences study design

All studies must disclose on these points even when the disclosure is negative.

Sample size	Sample sizes were determined based on preliminary data demonstrating statistically significant differences for each specific assay.
Data exclusions	In this study, no data were excluded from the analyses.
Replication	The replication of experiments in this study were described in the legend.
Randomization	Rice plants grown in the plant growth chambers or natural field conditions were randomized along with all required controls. The samples tested were randomized and replicated in all experiments.
Blinding	For data collection and analysis, the investigators were blinded to group allocation.

Reporting for specific materials, systems and methods

We require information from authors about some types of materials, experimental systems and methods used in many studies. Here, indicate whether each material, system or method listed is relevant to your study. If you are not sure if a list item applies to your research, read the appropriate section before selecting a response.

Materials & experimental systems

n/a	Involved in the study	
☐	☒	Antibodies
☒	☐	Eukaryotic cell lines
☒	☐	Palaeontology and archaeology
☒	☐	Animals and other organisms
☒	☐	Clinical data
☒	☐	Dual use research of concern
☐	☒	Plants

Methods

n/a	Involved in the study	
☒	☐	ChIP-seq
☒	☐	Flow cytometry
☒	☐	MRI-based neuroimaging

Antibodies

Antibodies used	anti-Ubi rabbit antibody (Beyotime, code number AF1705, 1:2000 dilution); anti-GST mouse antibody (Abmart, code number M20007S, 1:2500 dilution); anti-His mouse antibody (Abmart, code number M20001S, 1:2500 dilution); anti-MYC mouse antibody (Abmart, code number M20002S, 1:2500 dilution); anti-FLAG mouse antibody (Abmart, code number M20008M), 1:2500 dilution; anti-plant Actin mouse antibody (Abmart, code number M20009L, 1:2000 dilution); anti-OsSnRK1.1 antibody (prepared by Abmart Shanghai Co.,Ltd, 1:500 dilution)
Validation	For the commercial antibody, all validation statements can be found on the manufacturer' website.

Dual use research of concern

Policy information about dual use research of concern

Hazards

Could the accidental, deliberate or reckless misuse of agents or technologies generated in the work, or the application of information presented in the manuscript, pose a threat to:

No	Yes	
☒	☐	Public health
☒	☐	National security
☒	☐	Crops and/or livestock
☒	☐	Ecosystems
☒	☐	Any other significant area

Experiments of concern

Does the work involve any of these experiments of concern:

No	Yes	
☒	☐	Demonstrate how to render a vaccine ineffective
☒	☐	Confer resistance to therapeutically useful antibiotics or antiviral agents
☒	☐	Enhance the virulence of a pathogen or render a nonpathogen virulent
☒	☐	Increase transmissibility of a pathogen
☒	☐	Alter the host range of a pathogen
☒	☐	Enable evasion of diagnostic/detection modalities
☒	☐	Enable the weaponization of a biological agent or toxin
☒	☐	Any other potentially harmful combination of experiments and agents

Plants

Seed stocks	The rice cultivar Koshihikari, IR64, and Kongyu 131 were used as the WT control in this study, individually.
Novel plant genotypes	The present study does not involve novel plant genotypes.
Authentication	N/A
